# Supplementary material for: DMRforPairs: identifying Differentially Methylated Regions between unique samples using array based methylation profiles
Source: BMC Bioinformatics. 2014 May 15;15:141. doi: 10.1186/1471-2105-15-141 (PMC4046028; doi:10.1186/1471-2105-15-141)
Supplement: Additional file 2 — DMRforPairs output for the comparison of A431-MCF7 and NA17018-NA17105. Please start from the HTML files in each folder. Available via the BMC Bioinformatics website. [file 1471-2105-15-141-S2.zip › 1394847754114233_MOESM2_ESM/A431_MCF7/figures/37494.pdf]

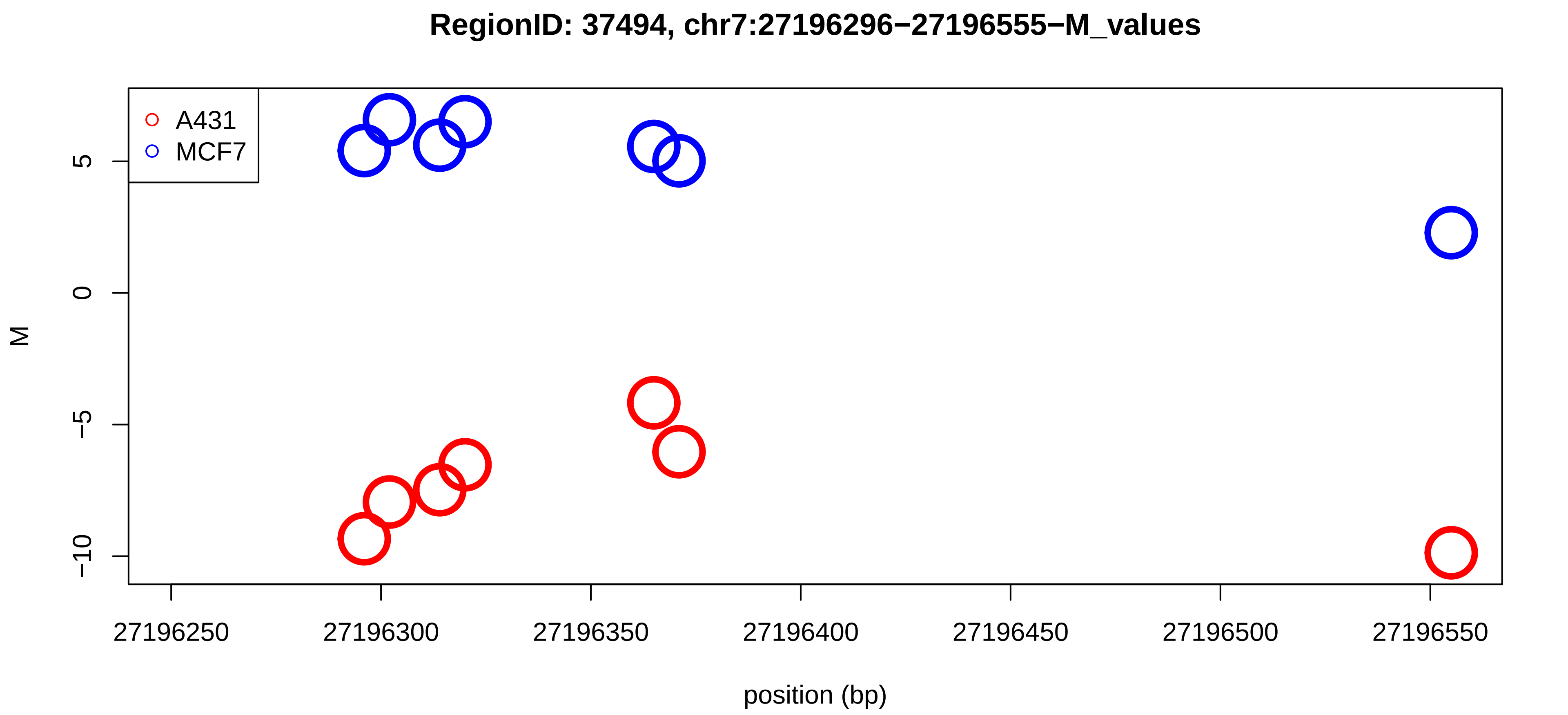
RegionID: 37494, chr7:27196296−27196555−M_values
A431
MCF7
M
5
0
−5
−10
27196250
27196300
27196350
27196400
27196450
27196500
27196550
position (bp)

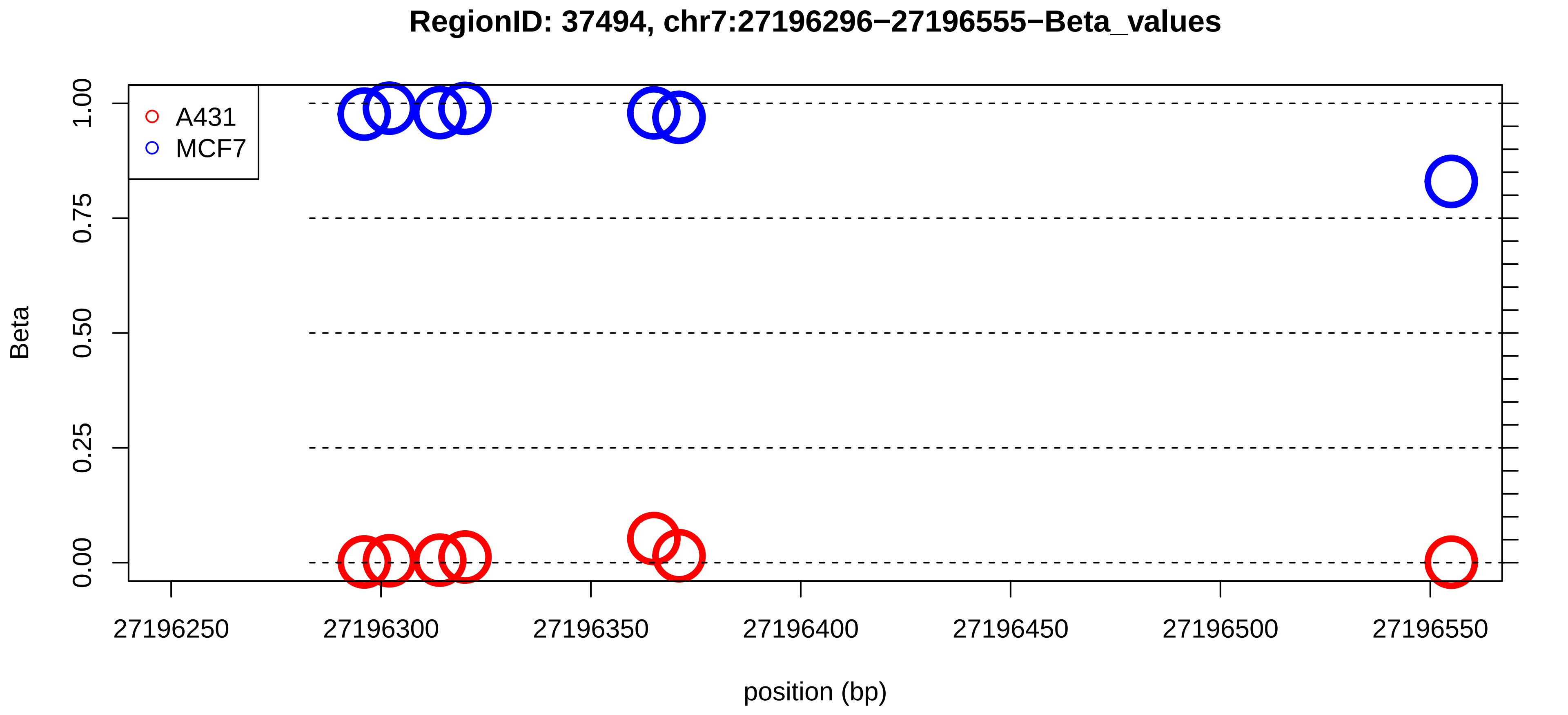
RegionID: 37494, chr7:27196296−27196555−Beta_values
A431
MCF7
Beta
1.00
0.75
0.50
0.25
0.00
27196250
27196300
27196350
27196400
27196450
27196500
27196550
position (bp)

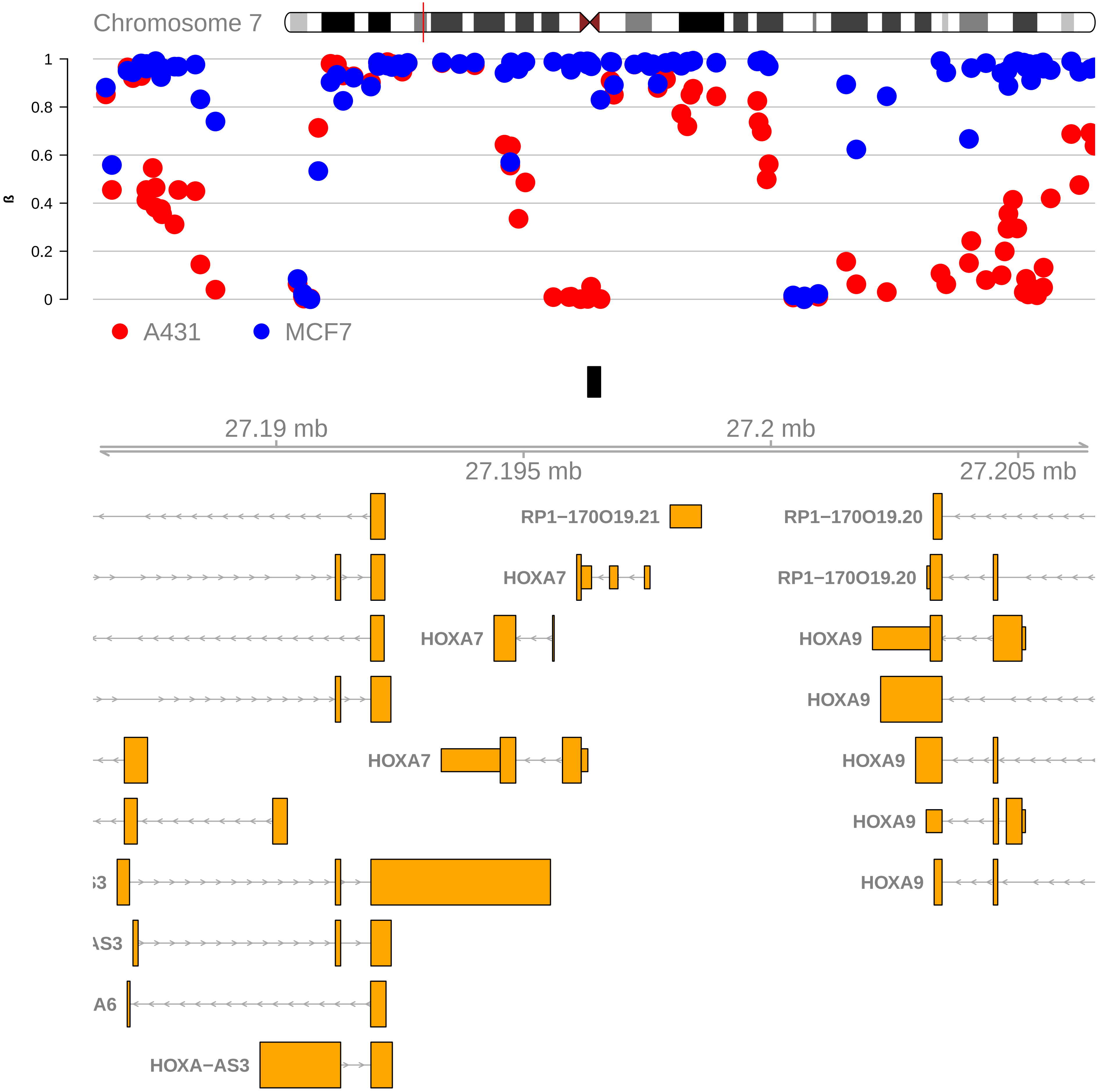

Chromosome 7
ß
1
0.8
0.6
0.4
0.2
0
A431
MCF7
27.19 mb
27.2 mb
27.195 mb
27.205 mb
RP1−170O19.21
RP1−170O19.20
HOXA7
RP1−170O19.20
HOXA7
HOXA9
HOXA9
HOXA7
HOXA9
HOXA9
HOXA9
HOXA−AS3
